# The Unplugged program in Chile (“Yo Sé Lo Que Quiero”) for substance use prevention among early adolescents: study protocol for a randomized controlled trial

**DOI:** 10.1186/s13063-021-05904-3

**Published:** 2022-01-25

**Authors:** Jorge Gaete, Saray Ramírez, Sofía Gana, Daniela Valenzuela, Ricardo Araya

**Affiliations:** 1 Universidad de los Andes, Research Center for Students Mental Health (ISME), Faculty of Education, Santiago, Chile; 2 Millennium Nucleus to Improve the Mental Health of Adolescents and Youths, Millennium Science Initiative, Santiago, Chile; 3 Doctoral Program in Developmental Science and Psychopathology, Faculty of Psychology, Universidad del Desarrollo, Santiago, Chile; 4 Department of Health Service & Population Research, King´s College London, London, UK; 5 David Goldberg Centre, Denmark Hill, London, UK

**Keywords:** Substance use, Adolescents, Prevention, Schools, Randomized control trial

## Abstract

**Background:**

Substance use is among the main contributors to disease among children and adolescents in the Americas region. The call for effective prevention of substance use among adolescents has resulted in numerous school-based programs, and particularly the Unplugged program has been proved to be successful in reducing the prevalence of different substances in seven European countries. The purpose of this study is to test the effectiveness of the Unplugged program in Chile (“Yo Sé Lo Que Quiero”).

**Methods:**

This is a cluster randomized controlled trial, parallel-group type, where “Yo Sé Lo Que Quiero” is compared to standard school preventive curricula in control schools. A total of 70 schools and 8400 adolescents are expected to be randomized with 1:1 allocation. During formative work, the Unplugged program was culturally adapted to Chile, and the instrument to assess the primary and secondary outcomes was validated. The effectiveness of this program will be assessed using the European Drug Addiction Prevention Trial Questionnaire (EU-Dap), measuring substance use prevalence and risk and protective factors in baseline, post-intervention, and four months after the end of the intervention.

**Discussion:**

The proposed study will be the first to test the effectiveness of a school-based substance use prevention program in Chile in a cluster randomized control trial and the first study evaluating the Unplugged program in Spanish-speaking Latin America. A model for disseminating the Unplugged program inside Europe already exists and has been implemented successfully in several countries. Thus, if the effects of the program are positive, wide implementation in Chile and Latin American countries is possible soon.

**Trial registration:**

ClinicalTrials.govNCT04236999. Registered on January 17, 2020.

## Administrative information

Note: the numbers in curly brackets in this protocol refer to SPIRIT checklist item numbers. The order of the items has been modified to group similar items (see http://www.equator-network.org/reporting-guidelines/spirit-2013-statement-defining-standard-protocol-items-for-clinical-trials/).

**Table:** 

Title {1}	The Unplugged program in Chile (“Yo Sé Lo Que Quiero”) for substance use prevention among early adolescents: study protocol for a Cluster Randomized Controlled Trial.
Trial registration {2a and 2b}.	Trial identifier NCT04236999 in Clinical Trials [ClinicalTrials.gov] under the registry name: “Unplugged, a Drug Use Prevention Program: Adaptation and Evaluation of Effectiveness Among Students in Chile”; [registered on 17-01-2020]
Protocol version {3}	Version 1 of 04-03-2021.
Funding {4}	This research is funded by the National Research and Development Agency [ANID]. Unique ID: Fondecyt Regular 1181724, and by ANID – Millennium Science Initiative Program – NCS2021_081.
Author details (5a}	J. Gaete: Research Center for Students Mental Health (ISME), Faculty of Education, Universidad de los Andes, Santiago, Chile. Millennium Nucleus to Improve the Mental Health of Adolescents and Youths, Millennium Science Initiative, Santiago, Chile. S.Ramirez: Research Center for Students Mental Health (ISME), Faculty of Education, Universidad de los Andes, Santiago, Chile. Millennium Nucleus to Improve the Mental Health of Adolescents and Youths, Millennium Science Initiative, Santiago, Chile. S. Gana: Doctoral Program in Developmental Science and Psychopathology, Faculty of Psychology, Universidad del Desarrollo, Santiago, Chile. Research Center for Students Mental Health (ISME), Faculty of Education, Universidad de los Andes, Santiago, Chile. Millennium Nucleus to Improve the Mental Health of Adolescents and Youths, Millennium Science Initiative, Santiago, Chile. D. Valenzuela: Research Center for Students Mental Health (ISME), Faculty of Education, Universidad de los Andes, Santiago, Chile. R. Araya: Department of Health Service & Population Research, King´s College London, London, United Kingdom.
Name and contact information for the trial sponsor {5b}	Investigator initiated clinical trial; Jorge Gaete (Principal Investigator). Contact Information: E-mail: jgaete@uandes.cl; Phone +56226182277; Address: Avenida Monseñor Álvaro del Portillo 12.455. Las Condes. Santiago, Chile
Role of sponsor {5c}	Funders played no role in the design of the study, collection, management, analysis, and interpretation of the report. They will not have ultimate authority over any of these activities.

## Introduction

### Background and rationale {6a}

Substance use and mental disorders are among the main contributors to disease among children and adolescents in the Americas region, representing 5.2% of the disability-adjusted life years (DALYs) and 17.2% of the years lived with a disability (YLD) in the population from 0 to 14 years [1]. In 2015, Chile led this region in tobacco use in the last month among adolescents in secondary education, reaching a prevalence of 23.7% [2]. Regarding the prevalence of marijuana use in the last year among adolescents in secondary education, Chile also led with 32.8% [2]. It should be noted that this last indicator has almost doubled in the last two decades from 14.8% in 2001 to 26.8% in 2019 [3]. Regarding the prevalence of alcohol use in the last month among secondary school adolescents, it has remained relatively stable during the last two decades, being 38.9% in 2011 and 29.8% in 2019 [3].

On the other hand, currently, the world is facing a COVID-19 pandemic, which has caused widespread disruptions in the lives of adolescents and their families, however to date, little is known about the pandemic’s unfolding impact on adolescent’s substance use [4]. It can only be mentioned one longitudinal study conducted in Iceland [4] where the results showed a decrease in substance use during the pandemic among adolescents as compared with pre-pandemic figures. The authors mentioned it might be attributed to an unintended benefit of the isolation during lockdowns.

Substance use among adolescents has been associated with the onset of psychiatric disorders such as depression and anxiety disorders [5, 6] in addition to dependency and substance abuse [6, 7], and it is known that the earlier in life the onset of substance consumption, the greater the risk of dependence in the future [8, 9]. The potential explanation of these findings is related to the crucial brain development on cognitive, behavioral, and mood functions that occur during adolescence [10–13].

The call for effective prevention of substance use among adolescents has resulted in numerous school-based programs developed for this purpose. For instance, a systematic review shows that the implementation of these programs has protective effects in the prevention of substance abuse [14]. A meta-analysis examining the effectiveness of interactive high school drug prevention programs on adolescent marijuana use in North America [15] shows that programs such as “Project ALERT,” “Life Skills Training,” and “All Stars” can potentially delay or prevent adolescent marijuana use. Additionally, a systematic review of European studies [16] highlighted another program called “Unplugged” implemented in seven countries, where positive results were obtained three months after the end of the intervention and 18 months later. Among these findings, there was a decrease in the frequency of drunkenness and cannabis use compared to controls [17].

Carrying out such research in Chile, where there is an urgent need for evidence-based prevention of substance use, is highly relevant. For example, nowadays, the government with the National Service for the Prevention and Rehabilitation of Drugs and Alcohol (SENDA) have a national program called “Elige Vivir Sin Drogas” in collaboration with the Icelandic Centre for Social Research and Analysis (ICSRA) and the application of the Planet Youth survey among adolescents attending year 10 [18]. This survey collects local information about risk and protective factors and substance use. Later, the results are presented to the local authorities, who have to design and implement preventive interventions. However, no randomized controlled trials (RCT) have been carried out in Chile to test the effectiveness of existing substance use prevention programs among adolescents. Therefore, without evidence-based preventive interventions, it will be difficult to change the current prevalence of this problem. The available programs in Chile are those developed, but not evaluated, by the government, such as “Continuo Preventivo,” which includes three programs “Descubriendo el gran Tesoro” (preschool students and parents/tutors), “Aprendemos a crecer” (1st to 4th grades students), and “La decision es nuestra” (7th to 12th grades students). In addition, there are a few independent initiatives such as the program “Familias Fuertes: Amor y límites,” developed by the Pan American Health Organization (PAHO), which has been implemented and evaluated in Chile [19], using a quasi-experimental design. This program intervened among adolescents and their parents in seven educational sessions. After six months, the results showed differences in parental styles but no change in reducing risk behaviors among adolescents.

The Unplugged program is a school-based intervention for the prevention of substance use in junior high schools, first implemented in seven European countries (Austria, Belgium, Germany, Greece, Italy, Spain, and Sweden). The Unplugged program targets students of 12–14 years old, and it has an aim to tackle both experimental and regular use of tobacco, alcohol, and illegal drugs. The curriculum is based on a comprehensive social influence approach, incorporating components of life skills into a Cognitive Social Influence model [20]. Special emphasis is given to the correction of normative beliefs about drugs and drug use. This program is organized in 12 sessions around three themes: (1) Knowledge and attitudes (1–4 sessions): students reflect on their knowledge on legal and illegal drugs, using tests to reach an objective assessment of this knowledge. These sessions also help students to make a distinction between desirable and undesirable consequences of taken drugs and between risk and protective factors. The program includes several interactive activities to facilitate the acquisition of this objective knowledge using several sources; (2) Intrapersonal skills (5–6 sessions): students reflect on normative beliefs, where perceived prevalence of drug use at students’ age is compared with the actual local prevalence, helping to develop critical thinking. Several role-play activities help to discuss peer-group attachment, group approach, peer-group allowance, and peer-group expectations. There are other interactive activities about emotion expression and positive ways to communicate and receive feedback; and (3) Interpersonal skills (7–12 sessions): students are instructed to practice refusal skills, assertiveness, and analyze coping strategies. They are also instructed and practice how to make decisions using a five-step model.

The effectiveness of this program was assessed by its authors over time; In 2010, the intervention was associated with a reduction in the prevalence of drunkenness episodes of around 38% and 26% in the use of marijuana in the last 30 days [17]. In 2011 it was shown to be associated with a decreased risk of reporting alcohol-related problems [21]. And in 2014, it was analyzed that after the intervention, there was a lower prevalence of drunkenness episodes and the intention to get drunk in students who attended schools with a low socioeconomic level [22].

In this study, we will adapt the Unplugged program for schools in Chile (henceforth in Chile “Yo Sé Lo Que Quiero,” YSLQQ), evaluating its effectiveness in this new context. Besides its huge practical significance, the project will contribute to the scientific discussion on the replicability of substance use school-based prevention programs in new contexts.

### Objectives {7}

The main objective of this study is to develop a culturally appropriate version of an evidence-based substance use prevention program called Unplugged, adapted to the Chilean culture (YSLQQ), and to test its effectiveness among early adolescents in primary schools in Santiago, Chile.

There are two stages in this study, formative work and the study of the effectiveness of the program. The specific objectives regarding formative work are the following: (1) To obtain the training in the Unplugged program from the developers. The approval for using the program in Chile is already granted, and the training of the research team was conducted at the beginning of the project (2018–2019); (2) To review the available Spanish version of the Unplugged program and culturally adapt it to Chile. This work was finished during 2019-2020; (3) To pilot the preliminary version of the cultural adaptation of the Unplugged program. This pilot acceptability and feasibility study were conducted in 2019, and the results are under analysis, and the article is in the writing process. (4) To pilot and validate the instruments that will be used to assess the effectiveness of the intervention. This validation was performed in 2018, and the results are submitted and under review. (5) Finally, the final stage is conducting a cluster randomized controlled trial to test the effectiveness of the program. The specific objective of this stage is (1) to compare the level of self-reported substance use of 6th and 7th graders participating in the YSLQQ program and control schools immediately after the end of the intervention and four months later, controlling by baseline assessment. This study will be carried out in 2022 and 2023, and we present its protocol here.

We hypothesize that at the end of the intervention, there will be a lower proportion of students who have initiated substance use and a lower proportion of students who have passed from experimental use to regular use of tobacco, alcohol, and cannabis in schools who received “Yo Sé Lo Que Quiero” when compared to standard school drug prevention curricula in control schools.

### Trial design {8}

This is a cluster randomized controlled trial, parallel-group type, where the YSLQQ program is compared to standard school prevention curricula in control schools. The standard school drug prevention curricula are delivered in the orientation class, henceforth “Treatment-As-Usual” (TAU) [23], and it will be used as a comparator to determine the superiority of YSLQQ over TAU in Chile. The patient allocation is randomized with a ratio of 1:1.

## Methods: Participants, interventions, and outcomes

### Study setting {9}

This trial will be performed in primary schools located in Santiago, Chile.

### Eligibility criteria {10}

#### Inclusion criteria for schools

- Schools located in Santiago (Chile)
- Schools having primary education (1st grade to 8th grade)
- Mixed-sex schools.
- Schools having at least two classes in the 6th and 7th grades.
- Schools with medium-high to high vulnerability (≥50%), measured with the School Vulnerability Index – National System of Equality Allocation (IVE-SINAE). This index is the proportion of students in a given school who have high vulnerability. This index considers the following socioeconomic variables to group the schools: mother’s educational level, father’s educational level, total monthly household income, among others [24].

#### Exclusion criteria for schools

- Schools having other manualized interventions for substance use prevention targeting the same grades.

#### Inclusion criteria for participants

- Attending 6th or 7th grade.

### Who will take informed consent? {26a}

Schools will be screened for eligibility to participate in this study based on the above-mentioned criteria. After the schools have been assessed as eligible by research assistants, the principal of the selected schools will receive study information. After the school authorities accept to participate in the study and sign a form, parents of students will be informed about the study and that the YSLQQ program will be part of the school curriculum sending information letters to their homes. Along with this information, the parent will receive the Informed Consent Form, and they need to sign it if they agree that their children participate in the study. Finally, the students will be informed about the study by research assistants and will be asked to sign an assent confirming their participation.

### Additional consent provisions for collection and use of participant data and biological specimens {26b}

Not applicable. No biological samples were collected.

## Interventions

### Explanation for the choice of comparators {6b}

The control group receives the standard school drug prevention curricula (“Treatment-As-Usual”, TAU) [23], which is not a manualized intervention. The standard curriculum includes a class called “Orientation” where students usually participate every week in a class where they receive teaching on health promotion and substance use preventive messages. Most of the material used in these classes comes from state agencies, such as the Ministry of Education and the National Service for the Prevention and Rehabilitation of Drug and Alcohol Abuse (SENDA). Moreover, the curriculum of this class usually does not follow a manualized intervention such as YSLQQ; therefore, we chose the usual orientation class as a treatment-as-usual comparator to determine the superiority of YSLQQ over the common practice in Chile.

### Intervention description {11a}

The intervention group will receive the “Yo Sé Lo Que Quiero” program. This program will be implemented during the academic year during school hours, mainly in the “Orientation” class. It has 12 1-h sessions taught once a week by teachers previously trained in a 3-day course. These 45-min 12 sessions are divided into three themes: (1) Knowledge and attitudes (sessions 1, 3, 5, and 9): students reflect on their knowledge of legal and illegal drugs, using tests to reach an objective assessment of this knowledge; (2) Intrapersonal skills (sessions 4, 6, 8, 10, 11, and 12): students reflect on normative beliefs, where perceived prevalence of drug use at students' age is compared with the actual local prevalence; and (3) Interpersonal skills (sessions 2 and 7): students are instructed to practice refusal skills, assertiveness, and analyze coping strategies. They also are instructed and practiced how to make decisions using a five-step model.

### Criteria for discontinuing or modifying allocated interventions {11b}

Students in the intervention group, as the program is part of the school curriculum, will participate in all sessions of the program. Even though they cannot leave the classroom if they do not want to participate in the sessions, they can leave the study at any time for any reason if they wish to do so without any consequences. This means that their information and collected data will not be analyzed. On the other hand, the control group will keep its condition during the whole trial.

### Strategies to improve adherence to interventions {11c}

Adherence to the “Yo Sé Lo Que Quiero” will be monitored by research assistants from the research team. Teachers will fill out a survey after each session informing the attendance of students and information about the quality of the delivery. Research assistants will be in close contact with school authorities and teachers, monitoring the progression of the program during study visits.

### Relevant concomitant care permitted or prohibited during the trial {11d}

The exclusion criterion states that manualized substance use prevention programs cannot be implemented in control schools. The aim of this study is to test the effectiveness of YSLQQ over TAU in Chile, and the common practice is not the implementation of manualized programs, as stated above in section [3].

### Provisions for post-trial care {30}

There is no potential harm or damage in this trial. The intervention group will receive an evidence-based intervention, and the control group will receive the usual prevention curriculum.

### Outcomes {12}

#### Primary outcome

1. Regular alcohol use measured with the European Drug Addiction Prevention Trial Questionnaire (EU-Dap) validated in Chile (manuscript under revision). Regular alcohol use is defined as the prevalence of any alcohol use during the last 30 days previous to the survey.

#### Secondary outcomes

1. Prevalence of substance use:

- Tobacco use: Tobacco use will be evaluated with two variables: 12-month tobacco use prevalence and 30-day tobacco use prevalence.
- Alcohol heavy use: Drunkenness will be evaluated with two variables: 12-month drunkenness prevalence and 30-day drunkenness prevalence.
- Marijuana: Marijuana use will be evaluated with two variables: 12-month marijuana use prevalence and 30-day marijuana use prevalence.

(2) Knowledge and skills promoted by the YSLQQ program:

- Positive and negative beliefs about tobacco use (no. of items = 8, e.g., How likely is that each of the following would happen to you if you smoke cigarettes in the next month? (v) Feel more relaxed; Answers 1= *Very unlikely* to 4= *Very likely*)
- Positive and negative beliefs about alcohol use (no. of items = 10, e.g., How likely is that each of the following would happen to you if you drink alcohol in the next month? (i) Do badly in school; Answers 1= *Very unlikely* to 4= *Very likely*).
- Positive and negative beliefs about marijuana use (no. of items = 10, e.g., How likely is that each of the following would happen to you if you take marijuana in the next month? (vi) Have more fun; Answers 1= *Very unlikely* to 4= *Very likely*).
- Positive and negative attitudes towards drugs (no. of items = 11, e.g., Here are some statements that people have made about illegal substances. How much do you agree with the following opinions on drugs? (iii) Using drugs is fun; Answers 1= *Strongly disagree* to 4= *Strongly agree*).
- Knowledge about substances (no. of items = 6, e.g. (i) Nicotine is the substance in cigarettes that causes lung cancer; Answers 1= *Yes*, 2= *No, 3= Don’t know*).
- Normative beliefs [perception of the number of user friends] (no. of items = 5, e.g., When you answer this question, think about the friends with whom you spend most of your leisure time. (iv) How many of them get drunk?; Answers 1= *None* to 5= *All of them*).
- Refusal skills (no. of items = 3, e.g. (i) You and your best friend are at a party where you meet new people, and you feel you really want to get to know them. Someone offers you to smoke hash together. Your friend accepts. Do you?; Answers 1= *Very unlikely* to 4= *Very likely*).
- School bonding (no. of items = 5, e.g., How much do you agree with the following descriptions of your school? (i) The students in my class enjoy being together; Answers 1= *Strongly disagree* to 4= *Strongly agree*).
- Decision-making skills (n. of items = 5, e.g., There are several possible ways to make decisions. How well do the following apply to you? (iv) I often regret something that I had decided; Answers 1= *Strongly disagree* to 4= *Strongly agree*).

### Participant timeline {13}

Table 1 shows the participant’s timeline.

**Table 1:**
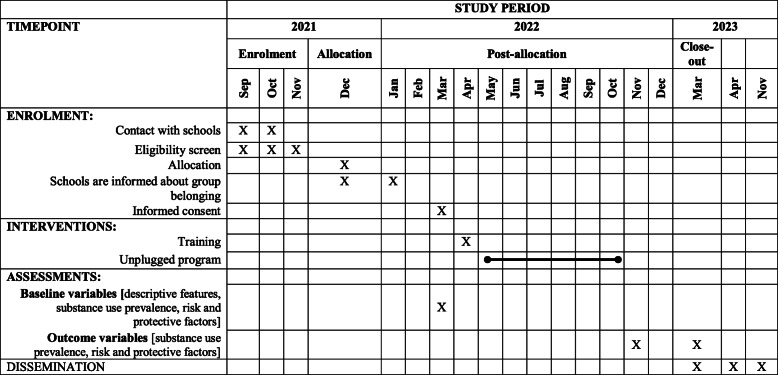
Standard Protocol Items: Recommendations for Interventional Trials (SPIRIT) diagram

### Sample size {14}

Assuming alpha 0.05 (two-sided), power 0.80, with a prevalence of regular alcohol use in the control arm of 4.18% (data available from a secondary analysis of Fourth National Survey of Violence in School Context 2014, which included this information for 7th and 8th graders), and assuming an Absolute Risk Reduction (ARR) of 2% in favor of the intervention group (38), 30 students per class, and Intraclass Correlation (ICC) of 0.02 (54), the estimated sample size is 4200 students per arm, corresponding to 35 schools in the intervention and 35 schools in the control group. Considering a potential refusal to participate of 40%, 120 schools fulfilling the inclusion criteria will be invited to participate. We used the “clustersampsi” command in Stata Software estimating the number of clusters and students in two arms, using the following command:

Clustersampsi, binomial samplesize p1 (X1) p2 (X2) m(y) rho(R)

where X1 = prevalence of regular alcohol use in arm 1, X2 = prevalence of regular alcohol use in arm 2, Y = number of children per school on average, and R = intracluster correlation. Finally, the two arms will be balanced to school size.

### Recruitment {15}

The strategies for achieving adequate school enrolment to reach the target sample size will include contacting and presenting the study municipality authorities who are expected to help to establish contact with school authorities. Additionally, research assistants will contact directly and inform school authorities about the purpose, requirements, and duration of the study. Our research team already has good collaborative networks with municipalities and schools in Chile.

## Assignment of interventions: allocation

Randomization will be performed once all schools are recruited. Schools will be randomly assigned to either group with a 1:1 allocation by computer-generated randomization.

### Sequence generation {16a}

Randomization will be performed once all schools are recruited. Schools will be randomly assigned to either group with a 1:1 allocation by computer-generated randomization.

### Concealment mechanism {16b}

After the randomization and allocation, schools will be informed by a research assistant of the group of belonging by email and confirmed by telephone. Each school will only receive their information of allocation. Additionally, this information will not be disclosed to the assessment research team (outcome evaluators) to keep the blind to the schools’ allocation.

### Implementation {16c}

An independent statistician will perform the randomization to assign to the study arms, and this statistician will give this information to a research assistant. Later, the research assistant will inform to schools by email and confirmed by telephone.

## Assignment of interventions: Blinding

### Who will be blinded {17a}

This is a double-blinded trial, blinded to the outcome evaluators and to the data analyst. Outcome evaluators will not be informed about the group of belonging and will be instructed not to ask for the school condition to the students nor to the school authorities. A data analyst will work with the final dataset where the group condition will be masked.

### Procedure for unblinding if needed {17b}

The design is open label with only outcome assessors and data analysts being blinded so unblinding will not occur. On the other hand, due to the nature of the intervention, the participants will know the allocation of the school.

## Data collection and management

### Plans for assessment and collection of outcomes {18a}

Data will be retrieved from a written self-reported questionnaire called the European Drug Addiction Prevention Trial Questionnaire [EU-Dap], and then the collected information will be transferred to an electronic database by trained research assistants.

The EU-Dap is a questionnaire aiming to screen substance use and to assess risk and protective factors among adolescents. It has 45 multi-item questions and focuses especially on tobacco, alcohol, and marijuana use. The EU-Dap instrument was developed by the research team of the Unplugged program in Europe. It was used to evaluate the Unplugged trial project, which took place between 2003 and 2005 in seven countries in Europe. In the Chilean validation primary analysis, all subscales had acceptable internal reliability (omega > 0.65). The original form of the questionnaire can be found at httpp:www.eudap.net.

### Plans to promote participant retention and complete follow-up {18b}

The schools, students, and their families will receive extensive information about the study setup and requirements during the recruitment. This information will include and stress the importance of completion of follow-up. From the start of the implementation of the assessments and the lessons of YSLQQ, students will be reminded of the value of their active participation during the whole project. Throughout the follow-up period, the researchers will check responses and, if necessary, contact schools and participants for completion of their follow-up.

### Data management {19}

After the participants have completed the questionnaires, the data will be entered into a secure platform without identifying information (each participant will be assigned an encrypted ID number). The original copies of the instruments will be filed and stored, under lock and key, in a self-storage, along with the list linking the participants’ names and ID numbers. Only the Principal Investigator, research assistants in charge of data entry, and the statistician will have access to the database. All people with access to the dataset will need to sign a Confidential Agreement to assure its commitment to not revealing identifying information.

### Confidentiality {27}

Research data will be stored using a study identification code for each participant. The key to the identification code list will only be available to the research team mentioned above and will be documented and safeguarded according to research guidelines after completion of the study. No participant identification details will be reported in publications or in any report of the study.

### Plans for collection, laboratory evaluation, and storage of biological specimens for genetic or molecular analysis in this trial/future use {33}

Not applicable. No biological specimens will be collected in this trial.

## Statistical methods

### Statistical methods for primary and secondary outcomes {20a}

General school features (size, number of teachers, etc.) will be used to compare participating schools with the ones that were invited but did not participate. Additionally, descriptive statistics will be used to compare the two arms at baseline.

Primary and secondary analysis will be conducted on an intention-to-treat basis. Odds ratios (OR) and their corresponding confidence intervals (95%CI) will be calculated as the measure of association between experimental condition (Intervention arm) and behavioral outcome, controlling for baseline outcome variable scores. Secondary analysis will be conducted considering adjustment for variables with marked imbalance at baseline. Additionally, secondary analyses will be made using the secondary outcomes and analyzed with the same approach. To take into account the hierarchical structure of the data and the cluster effect, a multilevel modeling approach will be followed in the analysis of the data. Data will be analyzed with Stata 17.0.

### Interim analyses {21b}

Not applicable. There will not be interim analyses because the data will be analyzed at the end of the trial.

### Methods for additional analyses (e.g., subgroup analyses) {20b}

Subgroup analyses will be performed to explore differences between males and females and between grades (6th and 7th grade). The statistical approach will follow the procedures described above.

### Methods in analysis to handle protocol non-adherence and any statistical methods to handle missing data {20c}

The primary outcome will be assessed using an intention-to-treat analysis. Missing data will be reduced to a minimum by using the appropriate measures: scourging students to fill out the whole questionnaire, a research assistant will revise the questionnaire when students end the evaluation and ask to complete the instruments if some questions are unanswered. Multiple imputations will be used to handle missing data in the primary and secondary analyses.

### Plans to give access to the full protocol, participant level-data and statistical code {31c}

The datasets produced during the current study will be available in an international database repository called UK Data Service.

## Oversight and monitoring

### Composition of the coordinating center and trial steering committee {5d}

This is a monocenter study designed, performed, and coordinated in Universidad de los Andes, Chile. Daily support for the trial is provided by the principal investigator, who takes supervision of the trial. Additionally, there is a data manager who organizes data collection and assures data quality. The study coordinator helped in trial registration and will coordinate study visits and reports. Study research assistants will help to identify potential participating schools, collect informed consents, ensure follow-up according to protocol, and resolve doubts from school staff or participants.

The study team will meet weekly during the whole duration of the study. There is no trial steering committee or stakeholder and public involvement group. The Ethical Scientific Committee of the Universidad de los Andes will check the presence and completeness of the investigation.

### Composition of the data monitoring committee, its role and reporting structure {21a}

A monitor from the Ethical Scientific Committee of the Universidad de los Andes will check once a year the presence and completeness of the investigation. This committee is independent of the sponsor and has no competing interests; further details about its charter can be asked via email: cec@uandes.cl.

### Adverse event reporting and harms {22}

The intervention or the data collection procedure does not infer harm among the participants. However, any situation that compromises the physical and psychological integrity of participants occurring during all different actions related to the project will be registered in a pre-design form. This information will be managed by the Principal Investigator and Project Coordinator, and, if necessary, they will contact school authorities, main caregivers, and local health providers. The whole research team will be trained in the Principles and the Code of Federal Regulations (CFR) for clinical research trials in the US [25] and will get the certification provided by the National Institute on Drug Abuse (NIDA). See https://gcp.nidatraining.org.

### Frequency and plans for auditing trial conduct {23}

A monitor from the Ethical Scientific Committee of the Universidad de los Andes will check once a year the presence and completeness of the investigation files, such as informed consents, inclusion and exclusion criteria, and data collection and storage.

### Plans for communicating important protocol amendments to relevant parties (e.g., trial participants, ethical committees) {25}

All substantial amendments will be notified to the ethics committee of the Universidad de los Andes. In case amendments concern or affect participants in any way, they will be informed about the changes. If needed, additional consent will be requested and registered. Also, online trial registries will be updated accordingly.

### Dissemination plans {31a}

The results of this research will be disclosed completely in international peer-reviewed journals. Both positive and negative results will be reported. An executive summary of the results will be given to school authorities.

## Discussion

The proposed study is the first to test the effectiveness of a school-based substance use prevention program in Chile in a cluster randomized controlled trial (cRCT), and the first study evaluating the Unplugged program in Spanish-speaking Latin American countries.

A model for disseminating the Unplugged program inside Europe already exists and has been implemented successfully in several countries. Thus, if the program effects are positive, wide implementation in Chile and other Latin American countries is possible in the near future. There are, however, some potential limitations. First, there might be an unknown impact of the COVID-19 pandemic on adolescents’ substance use, which may decrease substance use prevalence due to the isolation during lockdowns [4]. Second, there is a risk of low implementation quality in schools recruited into the cRCT. To avoid this threat, we will arrange face-to-face training sessions with the school personnel in order to motivate them to implement the Unplugged (YSLQQ) program as intended, as well as ongoing support and coaching during the implementation process. Finally, there might be a risk of difficulty in recruiting enough schools into the cRCT. To minimize this risk, we will prepare the recruitment carefully and inform the schools in good time, using the excellent networks of the members of the research team.

## Trial status

Recruiting will start in September 2021. The current protocol is version 1 of 04-08-2021. Patient recruitment is estimated to be completed around March 2022. Trial identifier NCT04236999 in Clinical Trials [ClinicalTrials.gov].

## Data Availability

The final trial dataset is planned to be available in an international data repository called UK Data Service.

## Abbreviations

EU-Dap: European Drug Addiction Prevention Trial Questionnaire
YSLQQ: Yo Sé Lo Que Quiero
cRCT: Cluster randomized clinical trial
DALYs: Disability-adjusted life years
YLD: Years lived with a disability
SENDA: National Service for the Prevention and Rehabilitation of Drugs and Alcohol
ICSRA: Icelandic Centre for Social Research and Analysis
PAHO: Pan American Health Organization
TAU: Treatment-As-Usual
